# Descriptive analysis of colorectal cancer in Zambia, Southern Africa using the National Cancer Disease Hospital Database

**DOI:** 10.11604/pamj.2018.30.248.12464

**Published:** 2018-08-06

**Authors:** Akwi Wasi Asombang, Richard Madsen, Michelo Simuyandi, Gilbert Phiri, Matthew Bechtold, Jamal Ahmad Ibdah, Kennedy Lishimpi, Lewis Banda

**Affiliations:** 1 Division of Gastroenterology, Warren Alpert Medical School of Brown University, Providence, Rhode Island, USA; 2 Department of Statistics, University of Missouri-Columbia School of Medicine, Missouri, USA; 3 Center of Infectious Disease Research in Zambia (CIDRZ), Lusaka, Zambia; 4 Cancer Disease Hospital (CDH), Lusaka, Zambia; 5 Division of Gastroenterology and Hepatology, University of Missouri-Columbia School of Medicine, Missouri, USA

**Keywords:** Colorectal cancer, non-communicable diseases, colon cancer, rectal cancer, cancer in Africa

## Abstract

**Introduction:**

Colon cancer is preventable. There is a plethora of data regarding epidemiology and screening guidelines, however this data is sparse from the African continent. Objective: we aim to evaluate the trends of colorectal cancer (CRC) in a native African population based on age at diagnosis, gender and stage at diagnosis.

**Methods:**

We conducted a retrospective analysis of the Cancer Disease Hospital (CDH) registry in Zambia, Southern Africa.

**Results:**

377 charts were identified in the CDH registry between 2007 and 2015, of which 234 were included in the final analysis. The mean age at diagnosis was 48.6 years and 62% are males. Using descriptive analysis for patterns: mode of diagnosis was surgical in 195 subjects (84%), histology adenocarcinoma in 225 (96.5%), most common location is rectum 124 (53%) followed by sigmoid 31 (13.4%), and cecum 26 (11%). 122 subjects (54%) were stage 4 at diagnosis. Using the Spearman rank correlation, we see no association between year and stage at diagnosis (p = 0.30) or year and age at diagnosis (p = 0.92).

**Conclusion:**

Colorectal cancer was diagnosed at a young age and late stage in the Zambian patients.

## Introduction

Colorectal cancer (CRC) is one of the most common preventable malignancies worldwide. According to Globocan 2012, it is the third most common cancer in men and second most common in women [1]. The highest rates are in developed nations and the lowest rates are presumed to be in Africa, except South Africa [1]. The age-standardized incidence rate in West Africa is 4.1/100,000; East Africa 6.5/100,000; Northern Africa 7.1/100,000; Southern Africa 10.9/100,000; South Africa 11.9/100,000; Mauritius 18.6/100,000; United States 25/100,000 and United Kingdom 30.2/100,000 [1]. The current colorectal cancer incidence and prevalence data from Africa are presumed to be an underestimate of disease burden. This underestimation is most likely related to data collecting methods, lack of centralized systems, limited knowledge amongst healthcare providers and patient-related factors such as late presentation. As noted, the highest rates of colorectal cancer within the African continent are in Southern Africa, which is where majority of the CRC African data originates (55 percent) [2]. Data suggests an overall decline of colorectal cancer, however an increase incidence in individuals younger than 50 years [3] and younger African Americans [4]. (11.9%) compared to whites (6.7%) [5] Zambia is a landlocked country in Southern Africa with a population of approximately 14 million. Colorectal cancer is the 6th most common cancer in Zambia, with an incidence rate of 4.8/100,000 [1], prevalence 3/100,000 and mortality rate of 3.8/100,000 [1]. GLOBOCAN data is obtained primarily from country cancer registries, which they acknowledge is not comprehensive, thus the true incidence and prevalence of colorectal cancer within the African continent is very uncertain. Further concerns about data validity include late patient presentation to health care providers and limited diagnostic capabilities and resources. With this in mind, we sought to analyze the current colorectal cancer database at the largest national cancer disease hospital in Zambia. We aimed to evaluate the trends of CRC in a native African population based on age at diagnosis, gender and stage at presentation.

## Methods

We conducted a retrospective analysis using the CDH database for CRC. CDH is the largest cancer hospital in the capital City (Lusaka) of Zambia and serves as the national referral center. CDH uses Microsoft access as its database, with data manually entered by an analyst. We identified all patients with colorectal cancer from inception of the CDH in 2006 until 2015. The following terminology was entered into the CDH access database to identify patients with CRC: “colon cancer” “rectal cancer” “colorectal cancer” “sigmoid cancer” “recto sigmoid cancer” “ascending colon cancer” “descending colon cancer.” We extracted the age, sex, race (white, black, other), year of colorectal cancer diagnosis and mortality. A data collection tool and master sheet code created a priori were used. Patient medical record numbers (MRN) identified in the registry were used to manually locate patient records. These records were manually reviewed by authors (AWA, LB) and the following was extracted: demographics (age, sex, race, place of residence and marital status), clinical presentation, date of diagnosis, mode of diagnosis, stage at diagnosis, treatment outcome and co-morbidity (HIV, Diabetes Mellitus, hypertension) and social (alcohol consumption, smoking). The AJCC (American Joint Committee on Cancer) guidelines were referenced for staging as follows [6]: stage 0 - carcinoma in situ/intramucosal carcinoma (involves lamina propria with no extension through the muscularis mucosa), stage 1- carcinoma involves the submucosa (through the muscularis mucosa but not into the muscularis propria) and without nodal involvement, stage 2 - carcinoma involves muscularis propria into or through the serosa, without nodal involvement, stage 3 - carcinoma into submucosa or through serosa with nodal involvement but no distant metastasis, stage 4: carcinoma with or without serosal involvement, with or without nodal involvement, with distant metastasis. Data was analyzed using SAS v9 (SAS Institute Inc., Cary, NC, USA). Descriptive statistics in the form of frequency distributions or sample means (age, BMI) and standard deviations as well as Spearman correlations for year/stage and year/age were found. Year and gender were compared using a test for trend. A p-value of less than 0.05 was used as level of significance. The study was approved by the institutional review board (IRB) at the University Of Missouri School Of Medicine and the Biomedical Research Ethics Committee of the University of Zambia School Of Medicine, Lusaka, Zambia.

## Results

**Figure 1:**
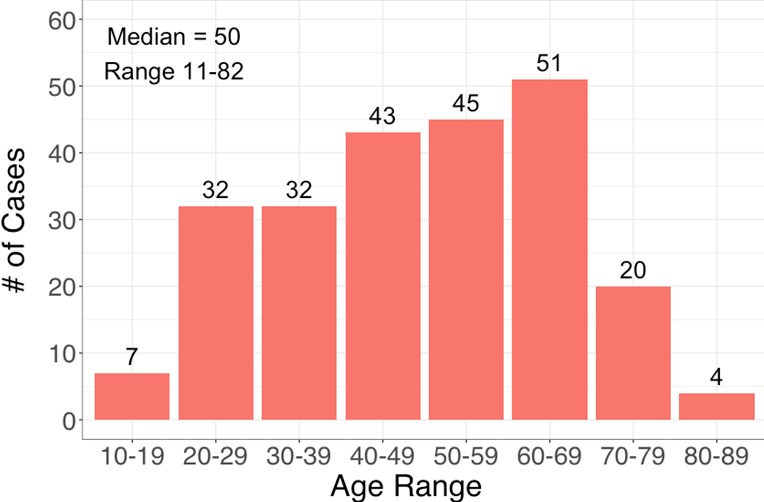
Age distribution of colorectal cancer in the CDH population

A total of 377 medical records were identified in the CDH database between 2007 and 2015, 33 charts could not be manually located, 110 excluded due to duplication (77) or non-CRC diagnosis (33), 234 were included in the final analysis. The mean age of diagnosis was 48.6 years and 62% are males (Table 1). The age at diagnosis ranges from 11-82 years with a median of 50 years and a mean of 48.6 years (Figure 1). Geographic location, defined as place of residence was identified in 229 (97.8%) CDH medical records. Geographically, 111 (48%) subjects resided in Lusaka and 1 (0.4%) outside of Zambia (Table 1). Co-morbidity and social factors were analyzed (Table 2), however meaningful conclusions could not be made due to significant missing information. The most common clinical presentation was hematochezia 88/213 (41.3%), followed by abdominal pain (59, 27.6%) and intestinal obstruction (56, 26.2%) (Table 3). The most common mode of diagnosis was surgical in 195 subjects (84%), followed by endoscopy (34, 14%) and imaging (4, 2%) (Table 4). The most common histology was adenocarcinoma in 225 (96.5%), most common location was rectum 124 (53%) followed by sigmoid 31 (13.4%) and cecum 26 (11%) (Table 4). Advanced disease (stage 4) at presentation was diagnosed 122 subjects (54%), followed by stage 3 (79, 35%), Stage 2 (19, 8%) and Stage 1 (1, 0.44%) (Table 5). Surgical management was the most common mode of treatment (26.6%) (Table 5). There was no statistically significant difference between both genders in location of tumour (p = 0.52) and stage at presentation (p = 0.27) (Table 6). A trend test shows no difference across years relative to the proportions of male/female (p = 0.56). Using the Spearman Rank correlation, we see no association between year and Stage at diagnosis (p = 0.30) or year and age at presentation (p=0.92).

**Table 1:** Baseline characteristics of the CDH Native African population

	N	Frequency (%)	Missing
**Sex**	234		0
Male	144	62	
Female	90	38	
**Mean Age (SD)**	48.5 (16.7)		
**Marital Status**	231		3
Single	37	16	
Married	166	72	
Widowed	21	9	
divorced	7	3	
**Residence**	229		5
Lusaka	111	48	
Central	20	8.7	
Copperbelt	45	19.6	
Northern	6	2.6	
Luapula	3	1.3	
Muchinga	4	1.75	
Eastern	11	4.8	
Northwestern	3	1.3	
Western	5	2.1	
Southern	20	8.7	
Out of country	1	0.4	

**Table 2:** Social factors, co-morbidity and BMI in CDH population

	N	%	Missing
**Smoking**			147
No	78	89.66	
Yes	9	10.34	
**Alcohol Consumption**			155
No	62	78.48	
Yes	17	21.52	
**Family History**			168
No	61	92.42	
Yes	5	7.58	
**History of HTN**			156
No	61	78.21	
Yes	17	21.79	
**History of DM**			161
No	66	90.41	
Yes	7	9.59	
**HIV Status**			163
No	49	69	
Yes	22	31	
**Mean BMI (SD)**	21.3 (5.1)		

CDH: Cancer Disease Hospital; BMI: Body Mass Index; HTN: Hypertension; DM: Diabetes Mellitus; HIV: Human Immunodeficiency Virus

**Table 3:** Clinical presentation at time of diagnosis in CDH population

	N	%	Missing
**Clinical Features**	213		21
Hematochezia	88	41.3	
Abdominal pain	59	27.6	
Intestinal Obstruction	56	26.2	
Constipation	38	17.8	
Rectal/anal pain	27	12.6	
Weight Loss	26	12.2	
Mass: anal/perianal/lower abdomen	11	5.1	
Abdominal Distension	12	5.6	
Diarrhea	11	5.1	
Vomiting	7	3.2	
Change in BMs	7	3.2	
Pain with BM	6	2.8	
Anal discharge	6	2.8	
Anemia	6	2.8	
Loss of Appetite	2	0.9	
Backache	2	0.94	
Melena	2	0.94	
Appendicitis	1	0.46	
Perforation	1	0.46	
Urinary incontinence	1	0.46	
Anal ulcer	1	0.46	
Fatigue	1	0.46	
tenesmus	1	0.46	

*The total adds to more than 100% since subjects may have presented with multiple features

**Table 4:** Clinic pathologic features

	N	%	Missing
**Mode of diagnosis**	**232**		2
Imaging	4	2	
Surgical	195	84	
Endoscopy	34	14	
**Histopathology**			1
Adenocarcinoma	225	96.57	
Sarcoma	3	1.29	
Carcinoid	2	0.86	
lymphoma	3	1.29	
**TUMOUR SITE**			3
Cecum	26	11	
Ascending colon	13	5.6	
Hepatic flexure	3	1.29	
Transverse colon	12	5.1	
Splenic flexure	5	2.16	
Descending colon	10	4.3	
Sigmoid	31	13.4	
Rectum	124	53.6	
Rectosigmoid	12	5	
Right	2	0.86	
left	1	0.43	

**Table 5:** Stage and treatment of CDH population

	N	%	Missing
**Stage**			9
Stage IA/IB	1	0.44	
Stage IIA/B	19	8.44	
Stage III	79	35.11	
Stage IV	122	54.22	
Not applicable	4	1.78	
**Treatment**	229		5
None	16	6.9	
Surgery	61	26.6	
Chemo-radiotherapy	23	10	
Palliative Radiotherapy	8	3	
Palliative Chemotherapy	14	6.1	
Chemotherapy	6	0.4	
Chemotherapy and surgery	53	23.1	
Chemo-radiotherapy and Surgery	34	14.8	
Palliative Surgery	4	1.7	
Palliative Chemo-radiotherapy	29	12.6	
Radiotherapy	5	0.02	

**Table 6:** Gender differences in tumor location and stage at diagnosis

	Male	Female	P
**Tumor Location**	**N (%)**	**N (%)**	0.52
Cecum	17 (11.97)	9 (10)	
Ascending Colon	9 (6)	3 (3)	
Hepatic Flexure	1 (.7)	2 (2)	
Transverse colon	10 (7)	2 (2)	
Splenic Flexure	4 (3)	1 (1)	
Descending Colon	6 (4)	4 (5)	
Sigmoid Colon	16 (11)	11 (12)	
Rectum	70 (49)	52 (58)	
Rectosigmoid	7 (5)	4 (5)	
Right Colon	2 (1)	0	
Left Colon	0	1 (1)	
**Stage**			0.27
I (A/B)	0	1 (1.15)	
II (A/B)	13 (9.7)	6 (6.9)	
III	43 (32)	36 (41.38)	
IV	78 (58.21)	44 (50.57)	

## Discussion

There is limited data regarding colorectal cancer in Zambia and sub-Saharan Africa. Our study is the first to evaluate the colorectal cancer trends, age of presentation and stage of diagnosis using Cancer Disease Hospital database. In a review of endoscopic and gastrointestinal pathology at a tertiary center in Zambia, Kelly et al identified 57 percent of colorectal cancer in individuals less than 45 years, higher than in the USA or UK, for reasons that were unclear [7]. Zyaambo and colleagues reviewed the distribution of cancers between 1990 and 2009, in Zambia, using the Zambian National Cancer registry [8]. Data in this cancer registry are collected from district hospitals (total number) using a standard cancer notification form [8]. They recognized the challenges of this database that result in incomplete or lower reported data. These challenges are attributed to logistical issues such as lack of an adequate postal service or transportation [8]. A rising trend in the incidence of colorectal cancer was noted in a retrospective analysis of colorectal cancer in the department of surgery at the University Teaching Hospital (UTH) in Zambia, comparing the years 1995-1996 to 2003-2004 [9]. In another year-long study, a single endoscopist's data of 85 patients revealed 28 percent with colorectal cancer and an age distribution between 14 years and 70 years [9]. Anecdotally, the Cancer Disease Hospital (CDH) in Zambia most commonly diagnoses patients with colorectal cancer between the age of 25 and 45 years. Understanding the basis for this age difference may reveal important clues to the etiopathogenesis of colorectal cancer in the Zambian population.

Our study provides evidence for younger age presentation of colorectal cancer and later stage of diagnosis at time of presentation. Our data also shows predominantly left sided (rectal cancer) in the native African population, which is different from the occurrence in African Americans who predominantly present with right-sided (proximal) colon cancer [4]. The right side predominance 60% versus 6% left sided has also been reported in Nigeria, West Africa, which is different from our study, however as in Zambia, the Nigerian population had a late stage at presentation and an increased incidence in the younger population, mean age 57 years [10]. In another study, Veruttipong et al compared the Egyptian population based cancer registry to the SEER database and found the incidence of colorectal cancer higher in the Egyptian population under the age of 40 years compared to the USA SEER population and predominant rectal lesion presentation similar to our Zambian CDH data [11]. This propensity for colorectal cancer in younger African population and left side predominance has also been supported in a South African population that compared within the different ethnic groups, revealing younger presentation in blacks (50 years) compared to whites (64 years) and Indians (60 years) and the most common cancer location is left sided (sigmoid and rectum) [12]. Limitations of our study are missing information in the co-morbidity and social factors limit interpretation, however highlights the importance of obtaining and recording a detailed medical history. The referral biases limit extrapolation to a general population-the increase number of cases noted in the capital city may have to do more with proximity to the Cancer Disease Hospital. The strengths of this study are that this is the first detailed analysis of colorectal cancer cases at CDH and in Zambia. Secondly the data contributes towards database strengthening, development of screening policies and serves as a platform for future research exploring risk factors.

## Conclusion

In conclusion, CRC accounts for a significant socioeconomic burden, mortality and morbidity hence it is important to raise awareness and implement preventative measures. Our study provides some baseline information for further studies evaluating risk factors and understanding of the development of colorectal cancer. To our knowledge, there are currently no colorectal cancer screening programs in most African countries. In a 2008 survey by the International Colorectal cancer screening network (ICRCSN) that included 35 programs from 24 countries, none were from the African continent [13]. Our research provides valuable data to understand the distribution of colorectal cancer and the development of policy in management and applicable screening guidelines.

### What is known about this topic

- There are racial differences in the occurrence of colorectal cancer;
- There is a trend for colorectal cancer occurrence in the younger age group;
- Colorectal cancer is preventable, yet most cases present at a later stage in Sub-Saharan African population compared to the Western world.

### What this study adds

- Our study suggests a late stage of colorectal cancer presentation in this population; there are currently no published colorectal cancer screening guidelines in Zambia, and to the best of our knowledge in Africa; colorectal cancer screening is an integral component to prevention, early detection and management to improve clinical outcomes hence our data supports exploring policy to develop colorectal screening guidelines;
- Our data shows an earlier age of presentation of colorectal cancer and importance for further research to understand this trend;
- Hematochezia is the most common clinical presentation hence further research exploring the use of screening tools such as the fecal occult blood test (FOBT) or the fecal immunochemical test (FIT) is warranted in this population.

## Competing interests

The authors declare no competing interest.

## References

[1] Ferlay J, Soerjomataram I, Dikshit R, Eser S, Mathers C, Rebelo M, Parkin DM, Forman D, Bray F (2015). Cancer incidence and mortality worldwide: sources, methods and major patterns in GLOBOCAN 2012. Int J Cancer.

[2] Graham A, Adeloye D, Grant L, Theodoratou E, Campbell H (2012). Estimating the incidence of colorectal cancer in Sub-Saharan Africa: a systematic analysis. J Glob Health.

[3] Siegel RL, Jemal A, Ward EM (2009). Increase in incidence of colorectal cancer among young men and women in the United States. Cancer Epidemiol Biomarkers Prev.

[4] Carethers JM (2014). Screening for colorectal cancer in African Americans: determinants and rationale for an earlier age to commence screening. Dig Dis Sci.

[5] Rahman R, Schmaltz C, Jackson CS, Simoes EJ, Jackson-Thompson J, Ibdah JA (2015). Increased risk for colorectal cancer under age 50 in racial and ethnic minorities living in the United States. Cancer Med.

[6] Wikipedia The Free Encyclopedia National Comprehensive Cancer Network (NCCN).

[7] Kelly P, Katema M, Amadi B, Zimba L, Aparicio S, Mudenda V, Baboo KS, Zulu I (2008). Gastrointestinal pathology in the University Teaching Hospital, Lusaka, Zambia: review of endoscopic and pathology records. Trans R Soc Trop Med Hyg.

[8] Zyambo C, Nzala S, Babaniyi O, Songolo P, Funkhouser E, Siziya S (2013). Distribution of cancers in Zambia: evidence from the Zambia national cancer registry (1990-2009). Journal of Public Health and Epidemiology.

[9] Kafwamfwa M (2010). Descriptive Study of The Pattern and Factors Associated With Colorectal Cancer in an HIV/AIDS era at University Teaching Hospital (UTH) Department of Surgery.

[10] Oribabor FO, Adebayo BO, Aladesanmi T, Akinola DO (2013). Anatomical Sites of Colorectal Cancer in a Semi-Urban Nigerian Hospital: Is There a True Rightward Shift. East Afr Med J.

[11] Veruttipong D, Soliman AS, Gilbert SF, Blachley TS, Hablas A, Ramadan M, Rozek L, Seifeldin IA (2012). Age distribution, polyps and rectal cancer in the Egyptian population-based cancer registry. World J Gastroenterol.

[12] Moolla Z, Madiba TE (2014). Trends in demographics and management of obstructing colorectal cancer. World J Surg.

[13] Benson VS, Atkin WS, Green J, Nadel MR, Patnick J, Smith RA, Villain P (2011). International Colorectal Cancer: screening network toward standardizing and reporting colorectal cancer screening indicators on an international level: the International Colorectal Cancer Screening Network. Int J Cancer.

